# Clinical outcomes with neoadjuvant versus adjuvant chemotherapy for triple negative breast cancer: A report from the National Cancer Database

**DOI:** 10.1371/journal.pone.0222358

**Published:** 2019-09-19

**Authors:** Nusayba A. Bagegni, Yu Tao, Foluso O. Ademuyiwa

**Affiliations:** Washington University School of Medicine, St. Louis, MO, United States of America; American Society for Investigative Pathology, UNITED STATES

## Abstract

**Purpose:**

Triple negative breast cancer (TNBC) patients frequently receive neoadjuvant chemotherapy (NAC). Only 50% will achieve pathological complete response (pCR). In this retrospective study, we evaluated TNBC outcomes with NAC vs. AC.

**Methods:**

Patients with stages II and III TNBC treated with NAC or AC between 2010 and 2013 were identified from the National Cancer Database. Baseline characteristics were compared with χ2 and two sample t tests. Kaplan-Meier survival analyses were computed in patients treated with NAC or AC, and log-rank tests used to examine differences. Unadjusted analyses of trends in proportions over time were performed using Cochran–Armitage tests. Log-binomial models were applied to estimate relative risks of non-pCR following NAC.

**Results:**

Of 19,151 patients, 5,621 (29.4%) received NAC, 13,530 (70.6%) received AC. NAC treated patients had worse OS compared to AC treated patients (73.4% vs. 76.8%; p<0.0001). pCR rate following NAC was 47.4%, and was associated with improved 5 year OS compared to non-pCR (86.2% vs. 62.3%; p<0.0001). In patients who received NAC, age, black race, clinical stage, diagnosis year, and Charlson-Deyo comorbidity score predicted non-pCR status. Use of NAC increased over the study period from 2010 to 2013 (27.8% - 31.2%; p = 0.0002).

**Conclusions:**

NAC may be inferior to AC in TNBC, likely related to the high frequency of non-pCR following NAC. It is unclear if removing the primary tumor prior to chemotherapy will have a beneficial biologic impact on therapeutic efficacy. These data should be considered hypothesis-generating as it is possible that the findings are due to selection bias, as physicians may use NAC for TNBC patients with more advanced local disease. Although, NAC still has a role in TNBC, developing biomarkers to identify patients likely to achieve pCR and benefit from NAC is an urgent need.

## Background

Breast cancer (BC) is the most commonly diagnosed malignancy in women. Approximately 266,120 new cases of invasive BC are estimated to be diagnosed in the United States in 2018 alone.[1] Although female BC is a leading cause of cancer mortality worldwide, it is often diagnosed at an early stage when it is potentially curable.[1] BC is biologically heterogeneous, with different subtypes exhibiting different prognoses. Gene expression profiling has identified five molecular subtypes with distinct behaviors and clinical outcomes: luminal A, luminal B, HER2 enriched, basal-like, and normal-like tumors.[2, 3] Basal-like tumors are predominately represented by the triple negative phenotype, characterized by the lack of estrogen receptor (ER), progesterone receptor (PR), and HER2/*neu* oncogene. Triple negative breast cancers (TNBC) account for 15–20% of all BC cases, are associated with a higher propensity for early recurrences, and have a worse 5 year overall survival (OS) compared with other BC subtypes.[4] In a study from the National Cancer Database (NCDB), TNBC patients had distinct demographic and racial/ethnic features as compared to non-TNBC patients.[5] TNBC is also associated with a higher frequency in younger premenopausal women, a more aggressive phenotype, including larger tumor size, higher tumor grade, but less likelihood of nodal involvement.[4, 6]

Although targeted therapies have improved management of ER-positive and HER2-positive BC, cytotoxic chemotherapy remains the mainstay of systemic therapy for TNBC patients. Despite worse prognosis, TNBC has higher response rates to chemotherapy.[7] Outcomes following neoadjuvant and adjuvant chemotherapy (AC) are comparable for BC patients in general.[8, 9] Achieving pathological complete response (pCR), commonly defined by the lack of invasive disease in the breast and axilla following neoadjuvant chemotherapy (NAC) (ypT0N0), serves as an early surrogate of long-term prognosis. NAC facilitates tumor downsizing for breast-conserving surgery, also allows for *in vivo* assessment of response to therapy, and provides prognostic information based on pathological response.[8] Another advantage of NAC is to allow early use of systemic therapy to eradicate occult distant micrometastatic disease without delay. Our prior single-institutional studies have suggested that NAC may be inferior to AC in TNBC in general, but is superior to AC only if pCR is achieved.[10, 11] Patients who do not achieve a pCR with NAC have worse outcomes with higher locoregional and systemic failure.[12–14] The administration of NAC has increased over time, with TNBC and HER2-positive patients having the highest increases over time.[15, 16] This study was designed to determine survival outcomes in TNBC patients treated with NAC vs. AC in a large United States oncology database. We also explore trends in the use of NAC for TNBC.

## Methods

Established in 1989, the NCDB is a joint national project of the Commission on Cancer of the American College of Surgeons and the American Cancer Society. This database is sourced from hospital registry data collected in Commission on Cancer accredited facilities. De-identified data provided by the NCDB was downloaded in May 2017. As NCDB data was not collected for the purposes of this study and is de-identified, this study was not considered to meet federal definitions under the jurisdiction of an Institutional Review Board, therefore an exemption was obtained. This article does not contain any studies with animals performed by any of the authors. All procedures performed in studies involving human participants were in accordance with the ethical standards of the institutional and/or national research committee and with the 1964 Helsinki declaration and its later amendments or comparable ethical standards. IRB approval not applicable as deemed not human subject’s research (HSR) by Washington University School of Medicine.

We performed a retrospective analysis of adult patients with AJCC7 clinical stages II and III TNBC who received systemic chemotherapy, and were diagnosed between 2010 and 2013. Only tumors that were known to be ER, PR, and HER2 negative were included as TNBC. HER2 status was recorded in the NCDB beginning in 2010, providing rationale for excluding patients diagnosed prior to 2010. We excluded patients diagnosed after 2013 to allow for at least 1 year survival period post diagnosis.

Demographic, socioeconomic, clinico-pathological features, treatment facility type, and geographic location, were included in the analyses. Treatment facility types were noted as follows: Community Cancer Program, Comprehensive Community Cancer Program, Academic/Research Program (includes NCI-designated comprehensive cancer centers), Integrated Network Cancer Program, or other/unknown types of cancer programs. These were grouped as academic programs (as defined by NCDB) vs. non-academic programs (all other treatment facility types not defined as academic/research program by NCDB). NCDB defines pCR as the absence of residual disease in the breast and lymph nodes following neoadjuvant chemotherapy.

Patients with non-invasive disease, stage I TNBC, distant metastases, inflammatory BC, missing treatment history or vital status (dead or alive), and patients who were not treated with chemotherapy were excluded. In order to ensure that only TNBC patients were represented, those who received treatment with hormone therapy or immunotherapy were excluded. Those with unknown pathological response status were also removed from the study population.

Chemotherapy timing was determined by identifying the sequence of systemic treatment and surgical procedures. Patients who received systemic therapy prior to surgery were defined as having received NAC. Patients who had surgery followed by systemic therapy were defined as having received AC. Patients who received both NAC and AC were also excluded. The median follow-up was the median time between diagnosis and the date of last contact or death.

Baseline sociodemographic and clinical characteristics across treatment type (NAC vs. AC) were evaluated by using Mantel-Haenszel χ2 test for categorical variables and two sample t test for continuous variables. The overall survival (OS) was defined as the interval between diagnosis and the date of death. OS by timing of chemotherapy receipt relative to surgery (NAC vs. AC) and by pCR status post NAC was assessed. Survival curves were estimated by the Kaplan-Meier method, and a two-sided P value of 0.05 from a log-rank test was considered a statistically significant difference. Five year OS rate (with 95% CI) was calculated by binomial method. The log-rank test was used to examine the statistical significance of the differences observed between the groups. Unadjusted analyses of trends in proportions over time were performed using Cochran–Armitage trend tests. Trends in receipt of NAC vs. AC were examined during each of the 4 years (2010–2013). Predictors of non-pCR after NAC, including age, race, income, education, insurance type, tumor grade, clinical stage, treatment facility type, diagnosis year, and Charlson-Deyo comorbidity score were assessed using a Log-binomial model and the results reported with adjusted relative risk of non-pCR and 95% confidence intervals (CI). 2-sided P<0.05 was considered statistically significant. All analyses were performed using SAS (version 9.4; SAS, Cary, NC).

## Results

**Fig 1:**
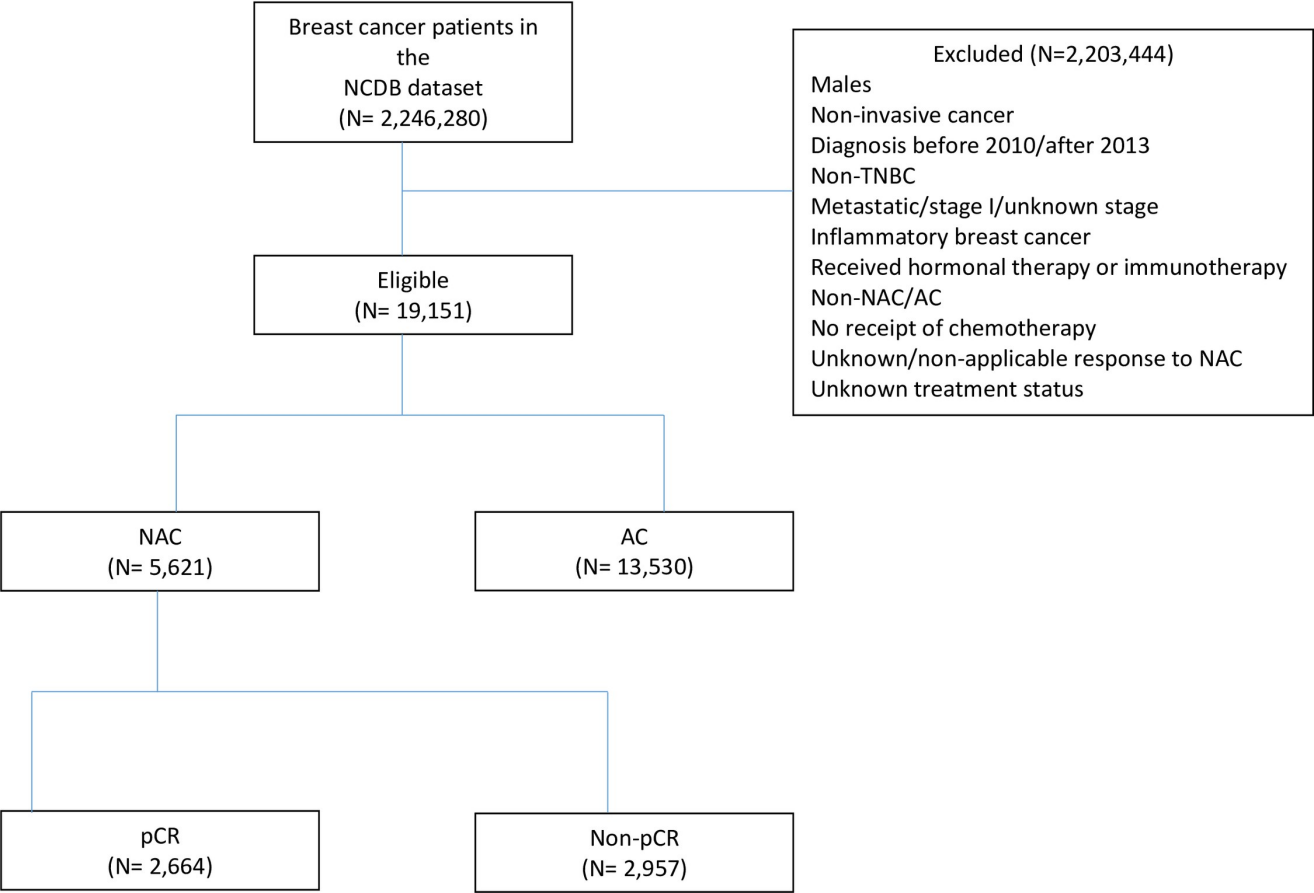
Patients with breast cancer in the NCDB data set included in the analytical cohort. **. Patients from the NCDB data set that were included in the analytical cohort.** Abbreviations: NCDB, National Cancer Database; TNBC, triple negative breast cancer; AC, adjuvant chemotherapy; NAC, neoadjuvant chemotherapy, pCR, pathologic complete response.

After all exclusions, 19,151 patients met the study inclusion criteria (Fig 1). The median age of the study population was 54 years (range: 21–90 years). 13,768 (71.9%) patients were White, and 4,463 (23.3%) were Black. 83.5% of had clinical stage II disease, and 16.5% had stage III disease. The majority of patients had poorly differentiated tumors (82.4%). Patient characteristics by timing of chemotherapy receipt (NAC vs. AC) are summarized in Table 1. Of patients receiving NAC, 68.4% had clinical stage II disease and 31.6% had stage III disease. The mean age of patients who received NAC was 51.9 years, vs. 55.7 years in those who received AC (p<0.0001). As compared with patients treated with AC, patients treated with NAC were more likely to have private insurance (64.3% vs. 58.2%) and live in a metropolitan area of the United States (88.2% vs. 84.1%). Patients were more likely to receive NAC at an academic program (33.4%), as compared to if treated at a non-academic program (25.4%) (p<0.0001).

**Table 1:** Patient characteristics according to treatment group.

Characteristic	Group NAC (N /%)	Group AC (N /%)	*P*-Value
Age, mean (SD)	51.9 (11.8)	55.7 (12.4)	<0.0001
Race	0.3874		
White	4007 (71.9)	9761 (72.6)	
Black	1343 (24.1)	3120 (23.2)	
Other	224 (4.0)	564 (4.2)	
Clinical Stage	<0.0001		
II	3843 (68.4)	12142 (89.7)	
III	1778 (31.6)	1388 (10.3)	
Grade	0.0001		
Well-differentiated	26 (0.5)	102 (0.8)	
Moderately-differentiated	649 (12.5)	1328 (10.4)	
Poorly-differentiated	4530 (87.0)	11391 (88.8)	
Charlson-Deyo Score	<0.0001		
0	4913 (87.4)	11128 (82.3)	
1	598 (10.6)	1953 (14.4)	
2	110 (2.0)	449 (3.3)	
Insurance Type	<0.0001		
No insurance	215(3.9)	454 (3.4)	
Private Insurance	3637 (64.3)	7780 (58.2)	
Medicaid	734 (13.2)	1427 (10.7)	
Medicare	911 (16.4)	3522 (26,3)	
Other Government	68 (1.2)	192 (1.4)	
Percentage with high school degree	<0.0001		
≥21%	953 (17.0)	2516 (18.6)	
13–20.9%	147 (26.3)	3728 (27.6)	
7–12.9%	1757 (31.4)	4272 (31.7)	
<7%	1418 (25.3)	2975 (22.1)	
Median Income Quartiles	<0.0001		
Less than $38,000	1006 (18.0)	2624 (19.5)	
$38,000 - $47,999	1186 (21.2)	3206 (23.8)	
$48,000 - $62,999	1515 (27.1)	3545 (26.3)	
≥ $63,000	1892 (33.8)	4111 (30.5)	
Geographic Location	<0.0001		
New England	238 (5.0)	559 (4.6)	
Middle Atlantic	595 (12.6)	1651 (13.5)	
South Atlantic	1329 (28.1)	3169 (26.0)	
East North Central	842 (17.8)	2377 (19.5)	
East South Central	292 (6.2)	988 (8.1)	
West North Central	343 (7.3)	821 (6.7)	
West South Central	391 (7.3)	900 (7.4)	
Mountain	207 (4.4)	547 (4.5)	
Pacific	488 (10.3)	1177 (9.7)	
Urban/Rural	<0.0001		
Metro	4842 (88.2)	11133 (84.1)	
Rural	70 (1.3)	235 (1.8)	
Urban	573 (10.5)	1865 (14.1)	
Treatment Facility Type	<0.0001		
Academic Program	1777 (37.6)	3547 (29.1)	
Non-Academic Program	2948 (62.4)	8642 (70.6)	
Diagnosis Year	0.0027		
2010	1332 (23.7)	3456 (25.5)	
2011	1459 (26.0)	3613 (26.7)	
2012	1381 (24.6)	3264 (24.1)	
2013	1449 (25.8)	3197 (23.6)	

Variables according to treatment group. Data are presented as No. (%) unless otherwise noted. Age as mean (standard deviation), defined in years. Abbreviations: NAC, neoadjuvant chemotherapy; AC, adjuvant chemotherapy; SD, standard deviation.

**Fig 2:**
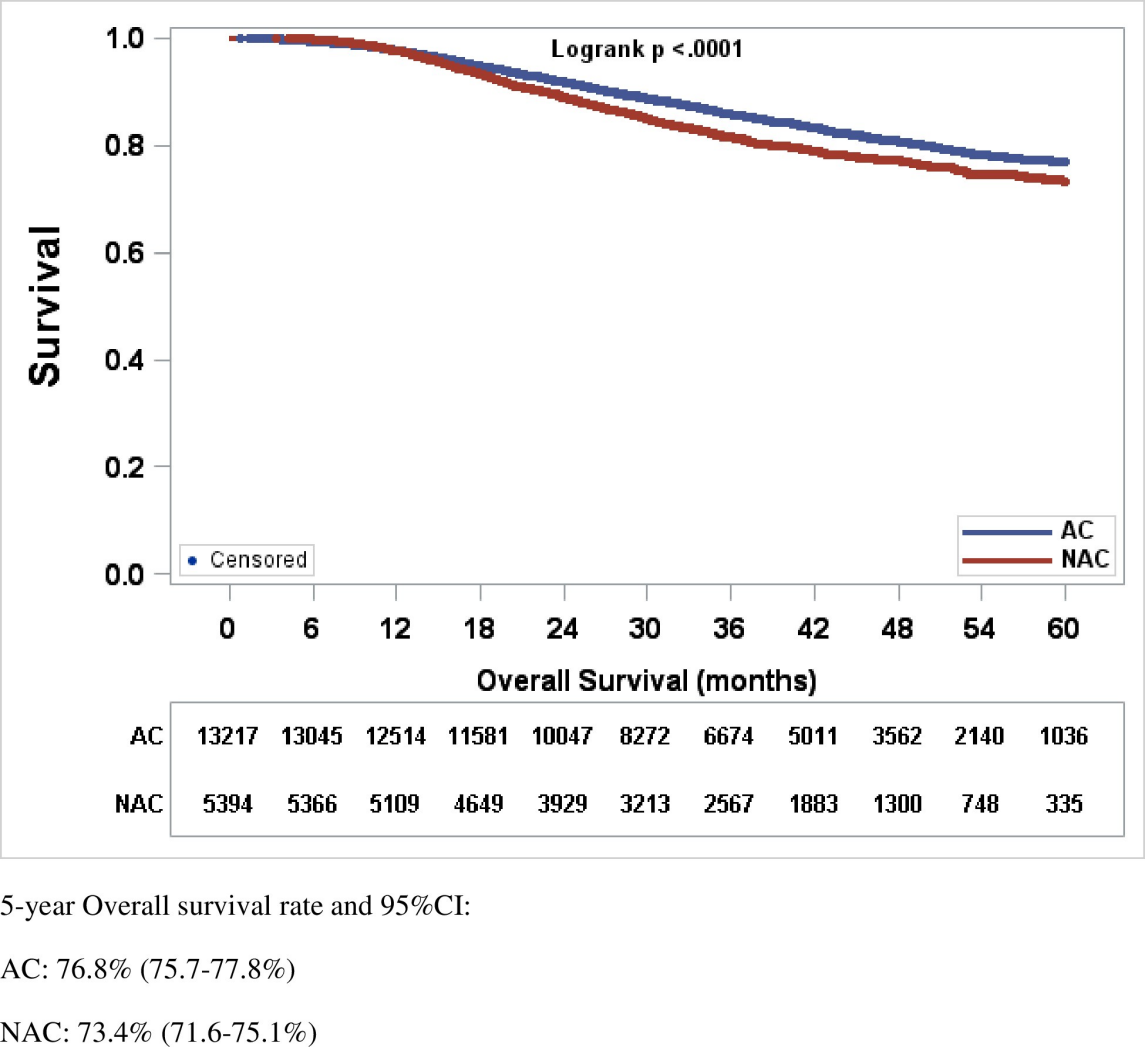
Kaplan-Meier estimates of overall survival in TNBC patients treated with NAC versus AC. OS rates presented as (%, 95% CI). OS rate was significantly superior for patients receiving AC versus NAC, 76.8% (75.5–77.8%) versus 73.4% (71.6–75.1%), *P*-value <0.0001. Abbreviations: CI, confidence interval; TNBC, triple negative breast cancer; OS, overall survival; AC, adjuvant chemotherapy; NAC, neoadjuvant chemotherapy.

**Fig 3:**
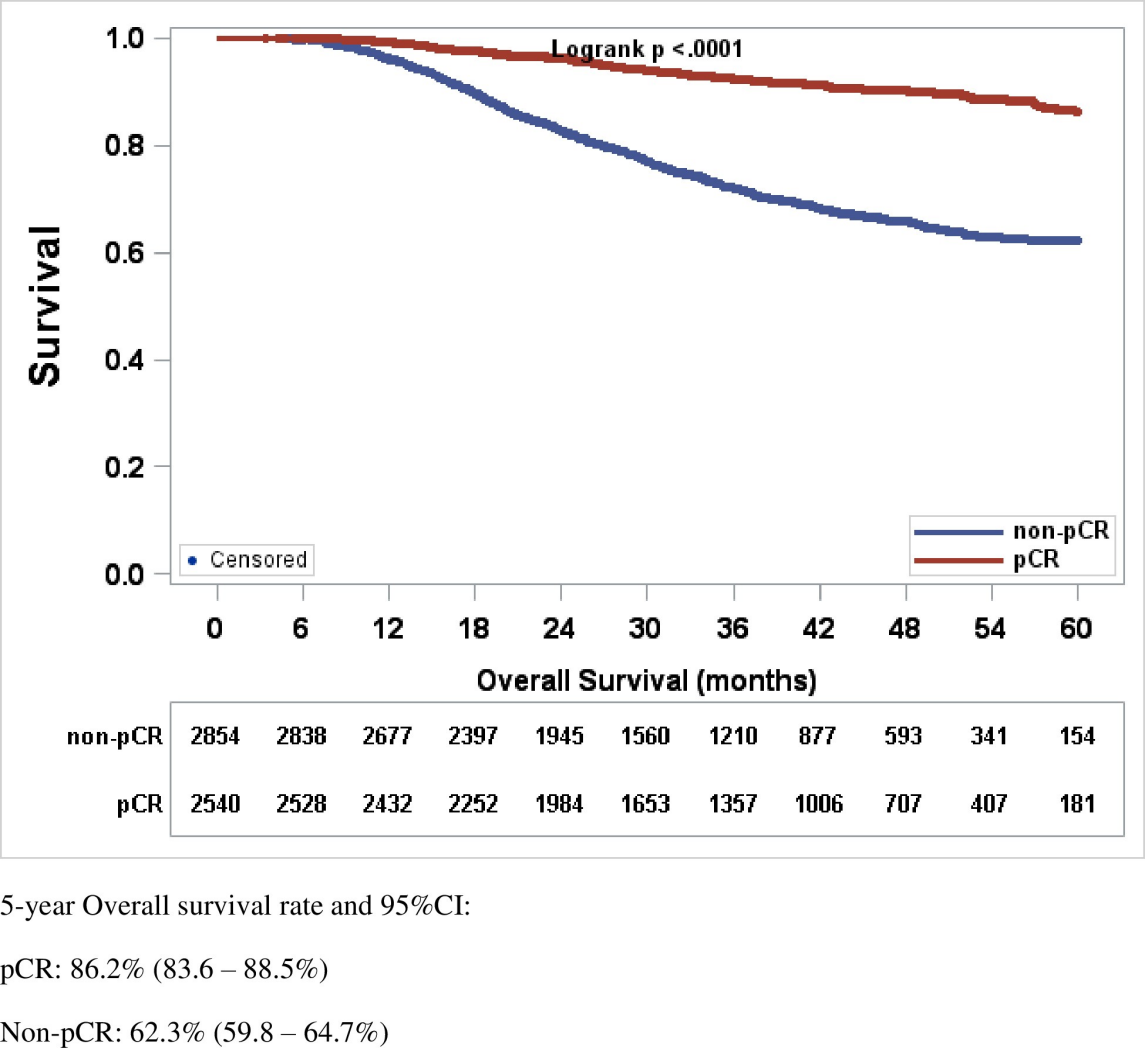
Kaplan-Meier estimates of overall survival in TNBC patients achieving pCR versus non-pCR following NAC. OS rates presented as (%, 95% CI). 5-year OS rate was significantly superior with pCR versus non-pCR following NAC in TNBC patients 86.2% (83.6–88.5%) versus 62.3% (59.8–64.7%), *P*-value <0.0001. Abbreviations: CI, confidence interval; TNBC, triple negative breast cancer; pCR, pathologic complete response.

At a median follow-up of 35.8 months, the 5 year OS of all patients was 75.8% (95% CI, 74.9–76.7%). OS was inferior in TNBC patients treated with NAC, than those treated with AC (73.4% [95% CI, 71.6–75.1%] vs. 76.8% [95% CI, 75.7–77.8%] p<0.0001) (Fig 2). Amongst the 5,621 patients treated with NAC, 2,664 achieved pCR (47.4%). Advanced age, Black race, and clinical stage III disease were all associated with a higher risk of having a non-pCR to NAC (Table 2). Achieving pCR following NAC was associated with significantly improved OS as compared to lack of pCR, with a 5 year OS rate of 86.2% (95% CI 83.6–88.5%) for pCR, vs. 62.3% (95% CI 59.8–64.7%) for non-pCR, respectively (p<0.0001) (Fig 3).

**Table 2:** Predictors of pathologic complete response following NAC.

Variable	No. events (pCR / total)	RR	95% CI
Age		1.009	1.006–1.012
Race	P = 0.044		
White	2054/4007	Ref	
Black	767/1343	1.087	1.025–1.153
Other	113/224	1.001	0.866–1.157
Grade	P <0.0001		
Well-differentiated	16/26	Ref	
Moderately-differentiated	409/649	1.008	0.742–1.369
Poorly-differentiated	2349/4490	0.868	0.641–1.175
Undifferentiated	16/40	0.745	0.465–1.196
Clinical Stage	<0.0001		
2	1888/3843	Ref	
3	1069/1778	1.175	1.115–1.238
Insurance Type	P = 0.9627		
No insurance	16/215	Ref	
Private insurance	1780/3637	0.899	0.789–1.024
Medicaid	421/734	1.015	0.881–1.168
Medicare	570/911	0.944	0.819–1.089
Other Government	34/68	0.869	0.650–1.159
Facility Type	P = 0.5575		
Non-academic	1589/2948	Ref	
Academic	947/1777	0.984	0.932–1.039
Diagnosis Year	P = 0.0082		
2010	746/1332	Ref	
2011	784/1459	0.958	0.895–1.026
2012	692/1381	0.902	0.839–0.968
2013	735/1449	0.923	0.859–0.991
Percentage with high school degree	P = 0.6151		
≥ 21%	516/953	Ref	
13–20.9%	797/1471	1.004	0.923–1.091
7–12.9%	916/1757	1.008	0.917–1.107
<7%	711/1418	1.000	0.896–1.117
Income	P = 0.4872		
Less than $38,000	558/1006	Ref	
$38,000 - $47,999	615/1185	0.984	0.902–1.075
$48,000 - $62,999	820/1515	1.063	0.971–1.164
≥ $63,000	947/1892	0.966	0.869–1.073
Charlson-Deyo Score	P = 0.0102		
0	2535/4913	Ref	
1	353/598	1.079	1.005–1.158
2	69/110	1.109	0.981–1.254

Variable that predict pCR in TNBC patients. Data presented as: Number of pCR events/total events, RR and 95% CI, as compared to the reference variable. Abbreviations: NAC, neoadjuvant chemotherapy; pCR, pathologic complete response RR, Relative risk; CI, confidence interval.

**Fig 4:**
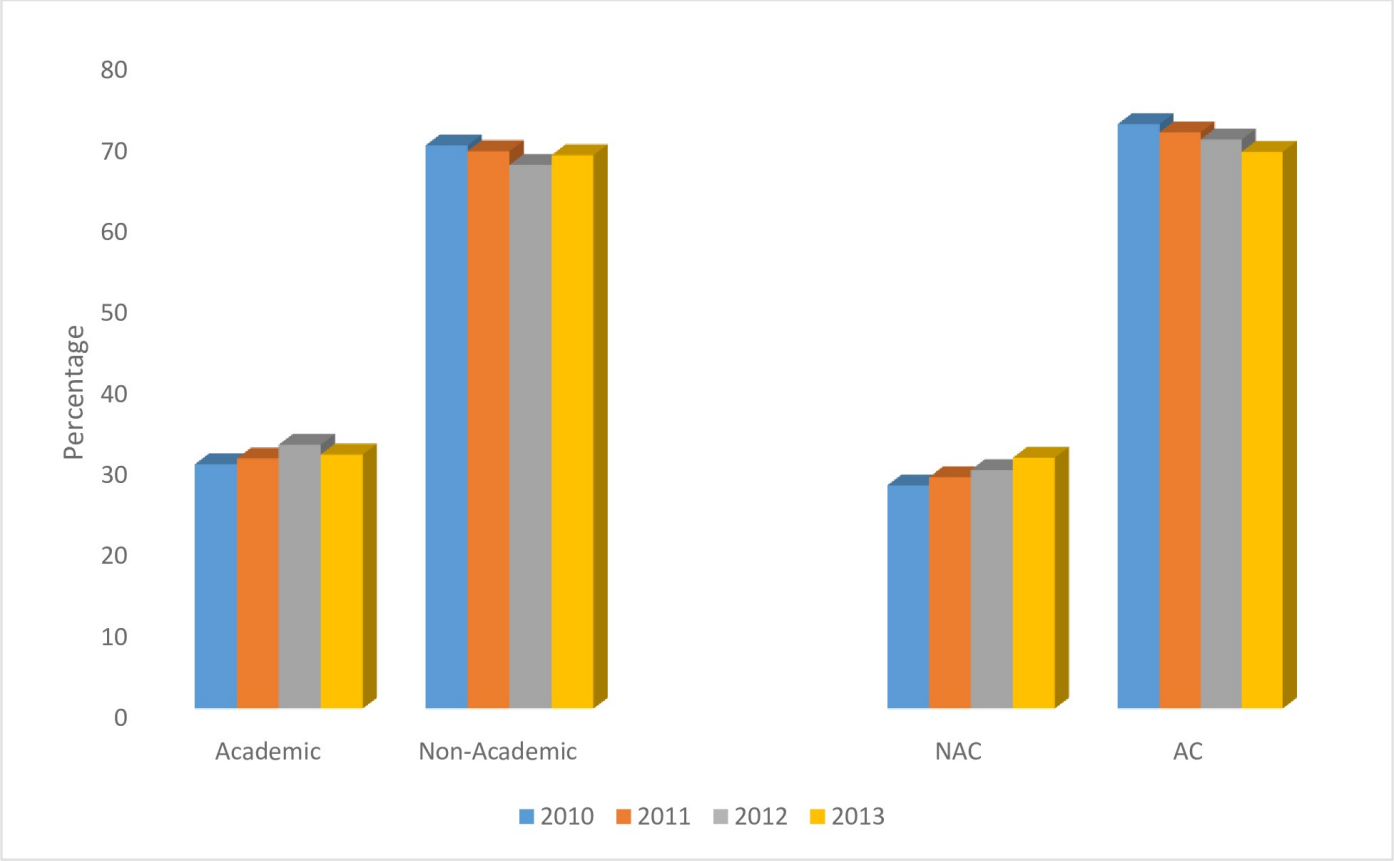
Trends in use of NAC and treatment facility type over time in TNBC patients. Cochrane-Armitage Trend test for treatment type (NAC vs AC) *P*-value 0.0002, and treatment facility type (academic vs non-academic program) *P*-value 0.095 used to determine trends over study period. Data presented by percent (%) of TNBC patients treated by diagnosis year (2010–2013) over time. Abbreviations: TNBC, triple negative breast cancer; NAC, neoadjuvant chemotherapy; AC, adjuvant chemotherapy.

Next, we evaluated time trends in timing of chemotherapy relative to surgery, and trends in setting of care. NAC use increased over the study period (27.8% in 2010, 28.8% in 2011, 29.7% in 2012, and 31.2% in 2013; p = 0.0002) (Fig 4). Receiving treatment at an academic program did not change during the study period (30.4% in 2010, 31.1% in 2011, 32.8% in 2012, and 31.6% in 2013, p = 0.095). There was a difference in 5 year OS between patients treated at an academic program as compared to a non-academic program, 77.0% vs. 74.3% (p = 0.003). In the subset of patients receiving NAC, there was no difference in 5year OS by treatment facility type (p = 0.063).

## Discussion

This study aimed to determine the overall survival for TNBC patients treated with NAC vs. AC in a multi-institutional cohort of United States breast cancer patients. To achieve this aim, we analyzed 19,151 TNBC patients from the NCDB. To our knowledge, this is the largest study evaluating the impact of chemotherapy timing on survival in TNBC patients treated with curative intent. Our study demonstrates that TNBC patients had a worse survival with NAC as compared to AC. These findings are in contrast to NSABP B-18, EORTC 10902, and the IBBGS, three large randomized trials evaluating NAC vs. AC.[8, 17, 18] These trials showed that there was no difference in survival among breast cancer patients receiving NAC compared to those receiving AC. However, there was no selection according to BC clinical subtype in these earlier studies. Other prior studies have shown conflicting results in outcomes specifically for TNBC patients treated with NAC vs. AC.[10, 19] Prior data from our institution show that NAC is superior to AC, only in TNBC patients who achieve pCR.[8] The pCR rate in this present study was 47.4%. This study confirmed a strong survival advantage with NAC in patients who achieve pCR also observed in other studies.[12, 20, 21] The differential outcome based on timing of chemotherapy relative to surgery is likely because of the high frequency of patients who did not achieve pCR with NAC seen in this study. TNBC patients who do not achieve pCR have a very high risk of disease relapse and subsequent BC-related deaths.

Another important observation is the long-term outcome in patients with pCR. TNBC and HER2-positive BC patients who achieve a pCR to NAC have a good prognosis.[12] The improvement in outcomes pCR patients relative to non-pCR patients in this current study is consistent with the improvements in OS and event-free survival in pCR patients in other studies.[22–24] Although pCR patients have superior outcomes than non-pCR patients, a number of those with pCR still have disease recurrence and BC-related deaths. There is no reliable method for detecting residual systemic microscopic disease after curative treatments, and to date, no risk factors for recurrence have been identified in either pCR or non-pCR patients. The potential to individualize management on the basis of prognostic markers remains an unmet need, and tools to predict disease recurrence are lacking. If patients who will recur due to chemotherapy resistance can be identified much earlier, innovative clinical trials can be developed, with a view to changing the natural history of recurrent TNBC.

Lastly, this study confirms the previously described increased trend in the use of NAC in TNBC patients.[16, 25] TNBC patients are more likely to respond to NAC than patients with ER-positive BC, thus NAC is now recommended as part of guidelines for managing TNBC patients with early-stage or locally-advanced disease.[26] Clinicians may be managing patients with more guideline-concordant care.[27] The rise in the number of neoadjuvant trials in TNBC is supported by the ability to assess *in vivo* responses to cancer therapeutics, and enables a platform for biomarker exploration. Thus, neoadjuvant trials have accelerated drug discovery, and have allowed clinicians to routinely administer NAC as standard of care. Achieving breast conservation by tumor downstaging, and improving cosmesis are also important advantages of NAC. Knowledge of pathological response to NAC also provides an opportunity to adjuvant therapy and enroll high-risk patients onto clinical trials.[28] Despite these numerous advantages of NAC over AC, there is a critical need to develop predictive biomarkers that can accurately identify patients likely to achieve pCR, and who are consequently better suited for NAC. Patients not likely to achieve pCR with standard NAC may then be enrolled onto innovative trials, be spared unnecessary side effects of ineffective cytotoxic chemotherapy, or avoid delaying definitive and potentially curative surgery.

This data must be interpreted in the light of its limitations. First, the analytic cohort was restricted to those diagnosed between 2010 and 2013. The NCDB started collecting HER-2 status in 2010; therefore, patients diagnosed prior to 2010 were excluded. This study period limits appreciating full trends in the use of NAC and long-term outcomes. Nonetheless, the median follow up of 3 years may allow an acceptable duration to evaluate outcomes in TNBC, as this subtype has a higher propensity for early recurrence following diagnosis. Second, lack of specific chemotherapy data is also another limitation of this study. However, since the study spanned a narrow, but contemporary period, we do not suspect that there were any substantial differences in the types of chemotherapy regimens utilized. Third, data regarding genomic testing (i.e. *BRCA* germline testing) were not available for analyses, however, these data do not impact outcome in TNBC patients receiving NAC vs. AC.[19, 29] Patients with BRCA-associated TNBC with chemoresistant disease may benefit from targeted treatments with agents such as PARP inhibitors as has been shown in the advanced setting.[30, 31] Fourth, retrospective analyses of observational data may be subjected to selection biases and unmeasured confounders. Immortal time bias is a limitation, as patients in the reference group had to be alive to receive AC, which would have been several weeks after those who received NAC. Fifth, although nodal status is prognostic in TNBC patients, it was not included in the analysis as accurate assessment of clinical nodal status and/or number of nodes involved from a registry type database may be inaccurate. In addition, accurate assessment of clinical nodal status and/or number of nodes prior to surgery may be inaccurate. Finally, it is now apparent that the molecular classification of TNBC also affects clinical outcome; unfortunately, this information is not used in routine clinical practice.[32, 33]

## Conclusions

In conclusion, multiple randomized trials show that NAC is equivalent to AC. Therefore, this current retrospective study suggesting that TNBC treated with NAC may have a worse outcome compared to AC, should only be considered as hypothesis-generating, and highlights the importance of determining biomarkers that can help predict those who will respond best to NAC by achieving a pCR.

## Abbreviations

NCDB: National Cancer Database
TNBC: triple negative breast cancer
BC: Breast cancer
NAC: neoadjuvant chemotherapy
AC: adjuvant chemotherapy
pCR: pathologic complete response
OS: overall survival
ER: estrogen receptor
PR: progesterone receptor
AJCC: American Joint Committee on Cancer
